# Repetitive Electric Stimulation Elicits Enduring Improvement of Sensorimotor Performance in Seniors

**DOI:** 10.1155/2010/690531

**Published:** 2010-04-14

**Authors:** Tobias Kalisch, Martin Tegenthoff, Hubert R. Dinse

**Affiliations:** ^1^Department of Neurology, Ruhr-University Bochum, BG-Kliniken Bergmannsheil, 44789 Bochum, Germany; ^2^Institute for Neuroinformatics, Neural Plasticity Laboratory, Ruhr-University Bochum, 44780 Bochum, Germany

## Abstract

Age-related changes occur on all stages of the human somatosensory pathway, thereby deteriorating tactile, haptic, and sensorimotor performance. However, recent studies show that age-related changes are not irreversible but treatable through peripheral stimulation paradigms based on neuroplasticity mechanisms. We here applied repetitive electric stimulation (rES) to the fingers on a bi-weekly basis for 4 weeks to induce enduring amelioration of age-related changes in healthy individuals aged 60–85 years. Tactile, haptic, and motor performance gradually improved over time of intervention. After termination of rES, tactile acuity recovered to baseline within 2 weeks, while the gains in haptic and motor performance were preserved for 2 weeks. Sham stimulation showed no comparable changes. Our data indicate that age-related decline of sensorimotor performance can be ameliorated by rES and can be stabilized by the repeated application. Thus, long-term application of rES appears as a prime candidate for maintaining sensorimotor functions in elderly individuals.

## 1. Introduction

Getting older is associated with a decline of cognitive and sensorimotor abilities. As for the sense of touch, age-related changes develop at all stages of the somatosensory processing pathway. Skin conformance is altered [1, 2], although mechanical properties of the glabrous skin might only have minor impact on discriminative abilities in elderly subjects [3, 4]. Besides morphological changes, the density of Meissner's and Pacinian corpuscles decreases in old age [5–9], while Merkel-neurite complexes appear to be less affected [6, 9–11]. Additionally, nerve conduction velocity (NCV) and sensory nerve action potentials (SNAP) slow down [12–16]. These changes are assumed to be due to an age-related reduction in the number and density of myelinated peripheral nerve fibers and a decrease in thickness of the myelin in the remaining fibers [17, 18]. There is evidence for substantial changes in gray matter density of the aged human CNS [19], with a nonlinear decline that is most rapid between 7 and 60 years, over dorsal frontal and parietal association cortices on lateral and interhemispheric surfaces [20]. Furthermore it was shown in PET and fMRI studies that prefrontal cortex activity tends to be less asymmetric in older than in younger adults [21]. Using multichannel EEG recordings in combination with electric source localization we have recently demonstrated that the size of the hand representation in the primary somatosensory cortex was increased in old subjects as compared to young adults in parallel with impaired tactile acuity [22]. 

Conceivably, the combined age-related alterations developing in the peripheral and central nervous system dramatically affect somatosensory information processing. For example, tactile acuity decreases in old age [4, 23–26], which is particularly severe in the more distal extremities [27, 28]. Interestingly, the impairment of tactile acuity is coupled with reduced dexterity scores as measured in a common pegboard test [25]. These results indicate that reduced acuity negatively affects fine motor performance in everyday activities such as grasping and handling small objects. Even simple activities of everyday life like buttoning a shirt or tying shoes are almost impossible when sensory information from the fingertips is corrupted. Recently we demonstrated the effects of mechanically shielding the fingertips on haptic and fine motor performance [29]. We found a significantly stronger impairment of elderly subjects' performance as compared to young subjects, indicating an increased need for tactile information in old age to achieve proper sensorimotor performance.

The typical approach to ameliorate age-related changes is to subject elderly individuals to intense schedules of training and practicing, and there is no doubt about the effectiveness of training-based intervention even at an advanced age [30–33]. Since many elderly individuals suffer from restricted mobility [34], however, additional and alternative approaches are needed that supplement and enhance, or even replace, conventional training procedures [35, 36]. 

Several years ago we introduced a paradigm that constitutes such an alternative approach to training: a specific form of repetitive sensory stimulation based on Hebbian synaptic plasticity to drive perceptual learning in young and adults [37]. In this paradigm, synchronous neural activity is evoked by tactile costimulation of small skin portions of the tip of the right index finger for a few hours. As a result, the finger representation in somatosensory cortex enlarged and tactile acuity improved [38–40]. In order to demonstrate that coactivation is mediated by established plasticity mechanisms, we tested its dependency on NMDA receptor activity. To scrutinize the role of NMDA receptors for activation-based plasticity, we used memantine, a substance known to block NMDA receptors [41]. We found that memantine eliminates coactivation-induced learning, both psychophysically and cortically. On the other hand application of neuromodulators such as amphetamine, which are used to support stroke rehabilitation by alteration of synaptic efficacy [42], resulted in almost a doubling of the normally observed improvement of tactile acuity and of cortical reorganization [40]. 

Recently we reported that this form of repetitive sensory stimulation is highly effective in elderly subjects as well [26, 36]. The unique advantage of the stimulation is its passive nature, that is, it does not require the active cooperation and involvement of the subject. Even more, attention is not required to drive plastic changes implying that the stimulation can be applied in parallel to other occupations and therefore might be substantially easier to implement and has a higher chance of being accepted as intervention. 

 In recent years we further optimized the paradigm in several respects. For example, the duration of application was reduced to 20 minutes [43] and the stimulation was extended from a single finger to all fingers of a hand [44]. In order to further explore the effectiveness and practicability of repetitive sensory stimulation, we here combine the advantage of short stimulation protocols and multifinger application with the advantages of electrical stimulation instead of using mechanical devices. Moreover, instead of a single application we here use a long-term treatment over 4 weeks. Our data show that under the described stimulation conditions, improvement of sensorimotor functions can be induced and maintained for at least 2 weeks.

## 2. Methods

### 2.1. Subjects

We investigated 7 right-handed elderly subjects (range: 66–79 years, mean age: 74.3 ± 4.4 years). In addition, 4 right-handed elderly subjects (range: 75–78 years, mean age: 76.5 ± 1.3 years) served as controls. There was no significant age difference between the subjects of both groups (*P* = .359). In all subjects, handedness was determined using the “Edinburgh Handedness Inventory” [45]. All subjects gave their written informed consent before participating. The study was approved by the local Ethics Committee of the Ruhr University of Bochum.

All elderly subjects were neurologically healthy, as assessed by a detailed questionnaire provided by a clinical neurologist. Individuals with polyneuropathy, peripheral nerve lesion or other neurological disorders were excluded from the study. Eligibility criteria were lucidity, independence in activities of daily living, absence of motor handicaps like functional impairment because of arthritis, or other causes of joint immobility. Furthermore, medication with central nervous effects in present or reported history was a criterion for exclusion. Tactile sensibility of the subjects' hands was checked prior to the experiments to test for neuropathies (cf. [26]). Additionally basic cognitive abilities were assessed using the “Mini Mental State Examination” [46].

### 2.2. Time Schedule

The time schedule of the experiments comprised of six weeks, with two interventions of repetitive electric stimulation (rES) per week (Tuesday, Thursday) for week 1 to week 4 and no intervention in week 5 and week 6. Measures of tactile, haptic, and fine motor performance were conducted before (*pre*) and after (*post*) the first interventions in week 1 and after every second intervention in weeks 2, -3, and -4 (*post-w2*, *post-w3*,* post-w4*). In week 5 and week 6 sensory and motor performances were assessed on every Thursday (*rec-w1* and *rec-w2*).

### 2.3. Repetitive Electric Stimulation (rES)

The rES was applied for 30 minutes per session. The rES-sequence was the same as described before [43] and consisted of stimulus-trains of 1 s (single pulse-duration: 0.2 ms (square), frequency: 20 Hz) and intertrain intervals of 5 s. The sequence was played back from a digital storage that triggered a standard two-channel TENS device (SM2-AKS, Pierenkemper, Germany) via a custom-made input-channel. Output channel 1 was used for stimulation of predominantly N. medianus-innervated fingers d1–d3 (thumb, index- and middle finger) and output channel 2 for predominantly N. ulnaris-innervated fingers d4 and d5 (ring- and little finger). The pulses were transmitted by adhesive surface electrodes (1∗4 cm, Pierenkemper, Germany) fixed on the first and third finger-segment (cathode proximal). Stimulation intensity was adjusted to the twofold sensory threshold resulting in an average initial stimulation intensity of 16.4 ± 1.8 mA on d1–d3 and 10.7 ± 2.4 mA on d4 and d5.

For sham stimulation in the subjects of the control group the electrodes were fixed but no current was transmitted during the interventions. Subjects were instructed that there was a subthreshold stimulation not creating any sensation.

### 2.4. Two-Point Discrimination

Spatial two-point discrimination thresholds were assessed on the tips of all fingers of the right hand using the method of constant stimuli as described previously [26, 35, 37–40, 44]. We tested seven pairs of brass needles; in addition, zero distance was tested with a single needle. To overcome problems in the use of two-point measurements associated with hand-held probes, we used a specifically designed apparatus that secures a standardized form of testing (cf. figures in [35, 36]). The apparatus allowed rapid switching between pairs of needles featuring different separations or one single needle (control condition). All tactile stimuli were applied to a fixed position on the skin of the fingertips for approximately 1s. According to own unpublished data, acuity thresholds obtained by gratings or by two-point measurements are largely equivalent (Pearson-correlation, *r* = 0.716, *P* ≤ .001, *n* = 22 subjects), although thresholds obtained by gratings are slightly lower in general. To account for the age-related decline in tactile acuity [23–26, 35, 36], we used larger needle separations for the elderly subjects (i.e., 1.5, 2.3, 3.1, 3.9, 4.7, 5.6, 7.0 mm) than usually used for young subjects (0.7–2.5 mm). The diameter of the needles was 0.7 mm and the diameter of the blunt endings was 200 *μ*m. Application-force was about 150 to 200 mN. Fixation of the tested fingers prevented the subjects from explorative finger movements. As described previously, test-retest reliability using this procedure was 0.90 for young subjects, and 0.88 for elderly subjects [36]. All eight-test conditions were presented eight times in randomized order resulting in 64 tests per session. The subjects, who were not informed about the ratio of needle-pairs and single needles (i.e., 7 : 1), had to decide immediately if they had the sensation of one or two needles. They were instructed to classify the percept of a single needle or doubtful stimuli as “one” but the distinct percept of two stimuli as “two.” The summed responses were plotted against the needle-distances resulting in a psychometric function, which was fitted by a binary logistic regression (SPSS; SPSS Inc., USA). Threshold was taken from the fit where 50% correct responses were reached. All subjects had to attend two training sessions to become familiar with the testing procedure before the assessment was finally started in the third session.

### 2.5. Haptic Object Recognition Test

The ability to recognize objects by explorative hand movements, that is, by haptic impression only, was tested by means of a custom-made visuohaptic test [26]. Conceivably, the identification of common objects depends massively on top-down information and is based on previous knowledge and former experience rather than manual exploration performance alone. To prevent the influence of previous knowledge and to create a comparable situation for all subjects, unfamiliar instead of common objects were used. The test consisted of five different groups of unfamiliar cubic objects (1.5 ∗ 2.7 ∗ 4.7 cm) made from common LEGO bricks (cf. figure in [35]). In each group, objects consisted of a specific number of rectangular bricks protruding on the sides in various positions. These constructional differences were highlighted by color to facilitate visual identification. One sample of each group was placed clearly visible on the desk in front of the subject. In a familiarization phase the subject was introduced in the structural features of the objects by the experimenter by unrestricted haptic and visual exploration. Afterwards the subjects were informed about the objective of the test: a total of 17 objects, hidden in a fabric sac, had to be explored by haptic perception only, that is, by explorative hand movements of the right hand. After the haptic exploration of one sample and coming to a decision about the group the object was assumed to belong to, it had to be placed in a box behind the specific sample on the desk. No visual verification during this process was allowed. After one initial training session all subjects indicated good comprehension of the test. Individual performance was assessed by measuring the time to fulfill the test and by counting the number of errors from three consecutive sessions. After each session the subjects received feedback, as every single object was checked according to their classification. Thereby the experimenter informed the subjects about possible errors. As the subjects were instructed to perform the test as fast and as accurate as possible, there is a tradeoff between speed and accuracy. Numerically, the large fluctuation in the standard deviations of the number of errors comes from the fact that some subjects make one or two errors, others perform with zero errors, which yield mean values in the range of one with large standard deviations, which therefore are much higher than typically found in the other test variables.

### 2.6. Pegboard Test

To test for fine motor performance we used a pegboard setup that is part of a commercial test-battery (MLS, Dr. G. Schuhfried GmbH, Mödling, Austria). The square pegboard (30∗30 cm) carries two rows of 25 small holes, one on the left side and one on the right side. Two containers, each equipped with 25 metal pegs, were placed in 30 cm distance from the right and left side of the board. The subjects were asked to pick the pegs with their right hand, one by one, from a container and insert them into the holes on the pegboard. If one of the metal pegs dropped during the transfer, they were instructed to go on with the next one. During the test the experimenter measured the time to complete the test and additionally the number of dropped pegs (i.e., errors). The test was performed in a standard version using long pegs (5∗0.25 cm) and in a demanding version using short pegs (1∗0.25 cm).

### 2.7. Statistical Analyses

To track improvement in spatial discrimination performance (thresholds of all fingers; d-prime values for data of all fingers), haptic performance (time to fulfill the test and number of errors), and fine motor performance (time to fulfill the test and number of errors for both versions of the test) we used repeated measures (rm) ANOVA with factors GROUP and SESSION, pairwise comparisons of single-session data were done by means of posthoc tests (one-sided Dunnett-tests with first session as reference and data < reference, Bonferroni and LSD). Additionally false alarm rates and hit-rates were calculated for data obtained in the two-point discrimination test and used for the evaluation of d-prime values (i.e., bias-free discrimination indices) [47]. For the calculation of these indices the false alarm rate was adjusted to 0.125 by default, if no false alarm was detected in a given session.

## 3. Results

### 3.1. Two-Point Discrimination

Baseline performance in two-point discrimination of all subjects was compared by means of two-tailed *t*-tests, showing no significant differences for thresholds of fingers d1 (target: 3.55 ± 0.30; control: 3.59 ± 0.31; *P* = .861), d2 (target: 3.52 ± 0.26; control: 3.63 ± 0.36; *P* = .566), d3 (target: 3.91 ± 0.19; control: 3.83 ± 0.10; *P* = .474), and d5 (target: 4.75 ± 0.32; control: 4.44 ± 0.20; *P* = .120). The thresholds of finger d4 differed significantly as subjects of the target group revealed higher thresholds compared to subjects of the control group (target: 4.41 ± 0.25; control: 4.08 ± 0.16; *P* = .041). 

**Figure 1:**
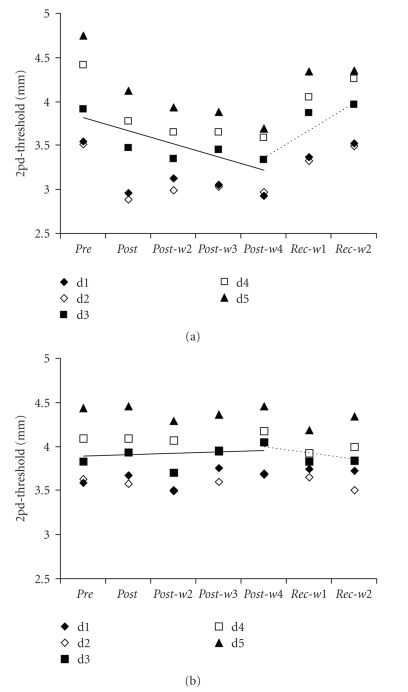
(a) Time course of the performance in the two-point discrimination (2pd) test. Two-point discrimination thresholds of all fingers of the dominant hand (d1=thumb; d5=little finger) averaged across subjects of the target group as obtained before (pre), during (post-w2–post-w4) and after the rES (rec-w1–rec-w2). There was a significant decrease in thresholds from pre session to post-w4 (Spearman correlation *r* ≥ −0.404, *P* ≤ .033), followed by a recovery of thresholds from post-w4 to rec-w2 (*r* ≥ 0.581, *P* ≤ .001). Solid line gives the linear regression for average thresholds of d1–d5 from pre session to post-w4 and dashed line gives the linear regression from post-w4 to rec-w2. (b) Same as (a) for the subjects of the control group, who received sham stimulation instead of rES.

The investigation of changes in two-point discrimination performance was conducted by means of rmANOVA for data obtained on every single finger of subjects in the target and control group. We found a significant interaction between the factors SESSION and GROUP for data obtained on d1 (*F*_(6,54)_ = 5.972, *P* ≤ .001), d2 (*F*_(6,54)_ = 6.672, *P* ≤ .001), d3 (*F*_(6,54)_ = 13.497, *P* ≤ .001), d4 (*F*_(6,54)_ = 10.192, *P* ≤ .001), and d5 (*F*_(6,54)_ = 9.270, *P* ≤ .001), with significant threshold changes only in the target group (Figure 1(a)) and not in the control group (Figure 1(b)). The analyses of rES-evoked improvement of the two-point discrimination performance by means of Dunnett posthoc tests revealed a significant reduction of thresholds of d1, d2 and d3 for sessions *post *(*P* ≤ .001), *post-w2 *(*P* ≤ .020), *post-w3 *(*P* ≤ .006) and *post-w4* (*P* ≤ .001) and a recovery of thresholds in session *rec-w1 *(*P* ≥ .116) and *rec-w2 *(*P* ≥ .774). The discrimination threshold of d4 was significantly reduced from the *post* to *rec-w1* session (*P* ≤ .007), and only recovered in *rec-w2 *(*P* = .290). For d5 we found a significant reduction of discrimination thresholds throughout all sessions from *post* to *rec-w2 *(*P* ≤ .013). For all tested fingers we found a significant decrease of discrimination thresholds from *pre* to *post-w4* session (Spearman-correlation: d1: *r* = −0.404, *P* = .033; d2: *r* = −0.417, *P* = .027; d3: *r* = −0.587, *P* ≤ .001; d4: *r* = −0.637, *P* ≤ .001; d5: *r* = −0.728, *P* ≤ .001; Figure 1(a)), with lowest thresholds in *post-w4* session (d1: 82.66 ± 3.14%; d2: 84.75 ± 4.84%; d3: 85.43 ± 4.95%; d4: 81.35 ± 6.17%; d5: 77.91 ± 7.31%). Starting from session *post-w4* there was a linear increase of discrimination thresholds (Spearman-correlation: d1: *r* = 0.581, *P* ≤ .001; d2: *r* = 0.712, *P* ≤ .001; d3: *r* = 0.758, *P* ≤ .001; d4: *r* = 0.770, *P* ≤ .001; d5: *r* = 0.655, *P* ≤ .001; Figure 1(a)), indicative of a recovery to initial performance level, culminating in thresholds of session *rec-w2* (d1: 99.60 ± 10.27%; d2: 99.65 ± 8.32%; d3: 101.67 ± 6.12%; d4: 96.68 ± 7.70%; d5: 91.95 ± 8.37%).

**Figure 2:**
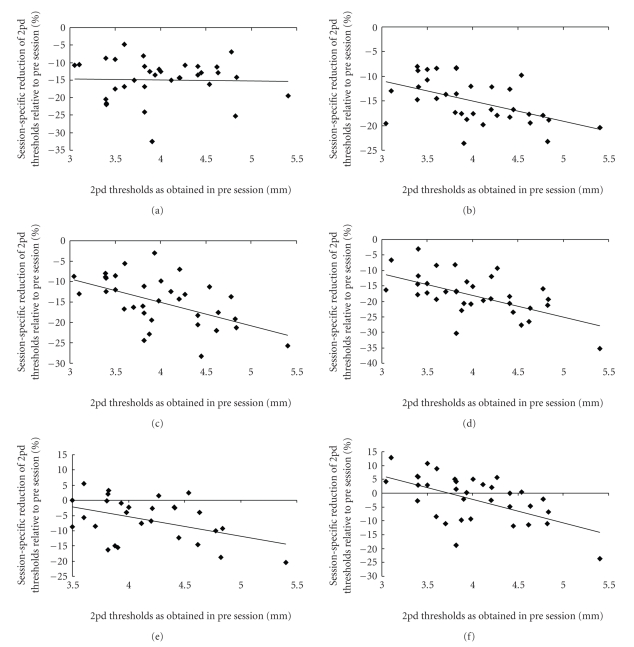
Gain in tactile acuity as a function of baseline performance. Plotted are percent changes (respective post session compared to pre session) in two-point discrimination (2pd) thresholds. (a) Although two-point discrimination thresholds of all fingers were reduced after the first application of rES (post), no correlation between initial threshold level and gain in performance was found (Pearson-correlation, *r* = −0.026, *P* = .884). In contrast, during succeeding sessions, a significant correlation emerged, which was maintained up to 2 weeks after termination of rES ((b) post-w2 (*r* = −0.499, *P* = .002); (c) post-w3 (*r* = −0.508, *P* = .002); (d) post-w4 (*r* = −0.545, *P* = .001); (e) rec-w1 (*r* = −0.461, *P* = .005); (f) rec-w2 (*r* = −0.530, *P* = .001)).

At any session of the experiment, except the *postsession*, we found a significant correlation between the initial two-point discrimination threshold of a finger (d1–d5) and the according percentage gain in performance (Pearson-correlations: *r* ≥ −0.461, *P* ≤ .005; *post* session: *r* = −0.026, *P* = .884). Correlations of initial performance level and session-specific improvement of single fingers are depicted in Figure 2. 

The described decrease of discrimination thresholds (Figures 1 and 2) in the first four weeks of the experiment (*post—post-w4*) and subsequent reversal in the fifth and sixth week (*rec-w1*—*rec-w2*) was accompanied by significant changes in d-prime discrimination indices of the subjects of the target group but not for subjects of the control group. There was a significant interaction of the factors SESSION and GROUP (repeated measures ANOVA) for averaged d-prime values of the subjects' right hand (*F*_(6,54)_ = 10.443, *P* ≤ .001). Averaged d-prime values of the subjects in the target group were 1.18 ± 0.08 in the *pre session*. After starting the intervention this value increased significantly (Dunnett posthoc test) to 1.26 ± 0.15 (*P* ≤ .001) in session *post*, 1.34 ± 0.05 (*P* ≤ .001) in *post-w2*, 1.34 ± 0.06 (*P* ≤ .001) in *post-w3,* and 1.41 ± 0.06 (*P* ≤ .001) in *post-w4*. Even during the *rec-w1* session we found an increased d-prime value of 1.18 ± 0.04 (*P* = .050). The initial discrimination index level was not reached until *rec-w2* (1.13 ± 0.06; *P* = .315).

Similar to the analyses of the absolute discrimination thresholds we observed a significant rise in discrimination performance, that is, increased averaged d-prime values, from session *pre* to *post-w4* by means of correlation analyses (Spearman-correlation: d1: *r* = 0.582, *P* ≤ .001; d2: *r* = 0.504, *P* = .006; d3: *r* = 0.640, *P* ≤ .001; d4: *r* = 0.667, *P* ≤ .001; d5: *r* = 0.552, *P*  =  .002). On the other hand there was no subsequent decrease of discrimination thresholds from *post-w4* to *rec-w2* (Spearman-correlation: d1: *r* = −0.643, *P* ≤ .001; d2: *r* = −0.677, *P* ≤ .001; d3: *r* = −0.800, *P* ≤ .001; d4: *r* = −0.761, *P* ≤ .001; d5: *r* = −0.546, *P* ≤ .001). 

### 3.2. Haptic Object Recognition Test

**Figure 3:**
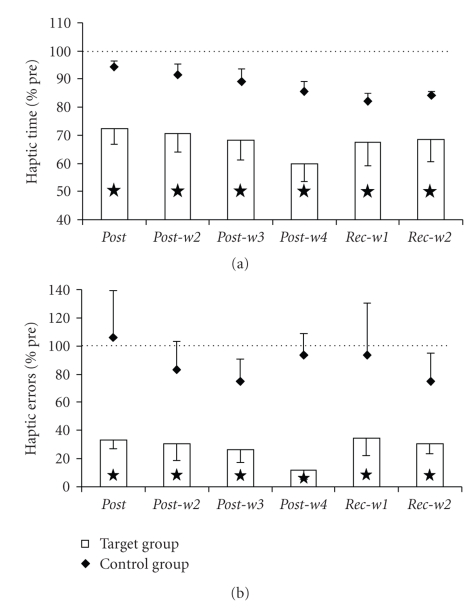
Haptic object recognition skills. (a) Percentage change of the time to fulfill the haptic object recognition test averaged across subjects of the target (bars) and control group (diamonds) relative to their performance in the presession. Stars indicate significant changes (*P* ≤ .05). (b) Same as (a) for the number of errors in the haptic object recognition test.

The ability to identify cubic arbitrary objects by haptic exploration was quantified by the average time to perform the test and the average numbers of errors occurring during testing. The baseline performance of subjects in both groups was compared by means of two-tailed *t*-tests. We found no significant differences in initial time to perform the test (target: 365.90 ± 50.24 s; control: 299.17 ± 81.41 s; *P* = .263) and initial number of errors (target: 2.71 ± 1.45; control: 1.25 ± 0.50; *P* = .102). There were significant interactions for factors SESSION and GROUP found in the analysis of time required to perform the haptic test (*F*_(6,54)_ = 3.555, *P* = .005). Significant changes in variable time were observed in subjects of the target group but not in the control group. In the target group the time to perform the test was reduced from 365.90 ± 54.24 s in the *presession* to 266.57 ± 79.46 s in *post* (LSD posthoc test, *P* = .041), 259.71 ± 79.46 s in *post-w2 *(*P* = .030), 250.24 ± 83.07 s in *post-w3 *(*P* = .019), 222.14 ± 84.39 s in *post-w4 *(*P* = .004), 251.76 ± 110.95 s in *rec-w1 *(*P* = .020) and 255.24 ± 108.46 s in *rec-w2 *(*P* = .024). In the control group the time to perform the haptic test did not deviate significantly from the *presession* (316.67 ± 84.25 s, *P* ≥ .270). Average performance (time) is depicted in Figure 3(a) for subjects of the control and the target group.

The rmANOVA of the number of errors during the performance of the test revealed group-specific differences. We found a significant interaction between the factors SESSION and GROUP for the variable number of errors in the haptic test (*F*_(6,54)_ = 3.674, *P* = .004), with significant changes only in the target group. In the target group the number of errors during the performance of the test decreased from 2.71 ± 1.45 in the *presession* to 1.00 ± 0.88 in *post* (Bonferroni posthoc test, *P* = .007), 1.00 ± 1.00 in *post-w2 *(*P* = .007), 0.67 ± 0.61 in *post-w3 *(*P* = .001), 0.24 ± 0.25 in *post-w4 *(*P* ≤ .001), 0.81 ± 0.50 in *rec-w1 *(*P* = .002), and 0.71 ± 0.40 in *rec-w2 *(*P* = .001). In the control group the number of errors in performance of the haptic test did not deviate significantly from the *presession *(1.33 ± 0.47, *P* ≥ 1.000). Average performance (errors) is depicted in Figure 3(b) for subjects of the control and the target group.

### 3.3. Pegboard Test

Fine motor performance of the subjects was investigated by means of a pegboard test. The baseline performance in the standard version of the test (using long pegs) showed no significant differences as calculated by two-tailed *t*-tests neither for the time to complete the test (target: 61.29 ± 19.42; control: 58.75 ± 6.56; *P* = .810) nor for number of errors (target: 0.00 ± 0.00; control: 0.13 ± 0.25; *P* = .200). In the data we found a tendency for group-specific differences for time (rmANOVA, SESSION∗GROUP, *F*_(6,54)_ = 2.259, *P* = .051). The posthoc test for data of the target group revealed a significant reduction for variable time from *presession* (61.29 ± 19.42 s) to *post*-*w4* (48.21 ± 6.59 s, LSD posthoc test, *P* = .023). Data obtained in all other sessions did not change significantly from the initial level (*P* ≥ .064). For the variable number of errors of the same test we found group-specific differences (rmANOVA, SESSION∗GROUP, *F*_(6,54)_ = 8.034, *P* ≤ .001). Subsequent analysis of data showed no significant changes in number of errors for data of the target group, as subjects had made no errors in the *pre session* (LSD posthoc test, *P* ≥ .091). Data of the control group on the other hand revealed a significant increase in number of errors from *pre session* (0.13 ± 0.25) to *post-w4 *(1.13 ± 0.48, LSD posthoc test, *P* = .023). All other sessions did not differ from initial performance (*P* ≥ .120).

**Figure 4:**
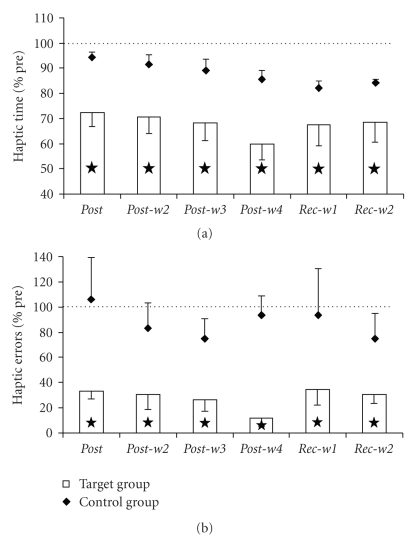
Fine motor performance in pegboard testing. (a) Percentage change of time to fulfill the pegboard test (short pegs) averaged across subjects of the target group (bars) and control group (diamonds) relative to their performance in the presession. Stars indicate significant changes (*P* ≤ .05). (b) Same as (a) for the number of errors in the pegboard test.

The baseline performance in the demanding version of the test (using short pegs) showed no significant differences as calculated by two-tailed *t*-tests neither for the time to complete the test (target: 95.93 ± 22.15; control: 98.75 ± 11.89; *P* = .821) nor for number of errors (target: 0.79 ± 0.27; control: 0.50 ± 0.00; *P* = .066). With regard to time we found significant differences between the performance of target group and control group (rmANOVA, SESSION∗GROUP, *F*_(6,54)_ = 2.330, *P* = .045), with significant changes only in the target group. The subsequent posthoc analyses of data obtained in subjects of the target group revealed a reduction of time needed from *presession* (95.93 ± 22.15 s) to *post-w3* (75.43 ± 13.76 s, LSD posthoc test, *P* = .029), *post-w4* (70.50 ± 12.25 s, *P* = .007) and *rec-w1* (75.57 ± 12.35 s, *P* = .030). The performance in sessions *post*, *post-w2* and *rec-w2* was on initial performance level (*P* ≥ .062) (Figure 4(a)). The investigation of the variable number of errors revealed no group-specific differences (rmANOVA, SESSION∗GROUP, *F*_(6,54)_ = 1.680, *P* = .144). The posthoc test calculated on data of the target group showed a significant reduction in number of errors from *presession* (0.79 ± 0.27) to *post* (0.21 ± 0.27, LSD posthoc test, *P* = .008), *post-w2* (0.14 ± 0.24, *P* = .003), *post-w3* (0.14 ± 0.24, *P* = .003), and *post-w4* (0.21 ± 0.39, *P* = .008). Performance in *rec-w1* and *rec-w2* was on initial level (*P* ≥ .090). The performance of subjects in the control group did not differ significantly from *presession* (0.5 ± 0.0, LSD posthoc test, *P* ≥ .175) (Figure 4(b)).

## 4. Discussion

Here we demonstrate that repetitive electric stimulation (rES) applied to all fingers of a hand on a bi-weekly basis for 4 weeks evoked an improvement of sensorimotor performance in neurologically healthy seniors aged 60 to 85 years that was maintained for up to 2 weeks after the end of intervention. On average, tactile acuity of the fingertips as estimated by two-point discrimination thresholds was significantly improved for one week after the last application of rES. Tactile acuity of individual fingers showed an even longer lasting improvement up to two weeks. Generally, the improvement of tactile performance accumulated gradually over time and was also apparent in bias-free discrimination indices. We found a baseline-dependency in all sessions demonstrating an inverse relation of initial performance and improvement after rES. In addition, haptic performance was significantly improved after repeated application of rES, and the gain was maintained by the subjects within the observation window up to 2 weeks after termination of rES. Finally, fine motor performance of the subjects was improved depending on the difficulty level of the test, with minor improvement in the standard test, but strong improvement in the demanding test. Performance went back to initial performance levels within one week.

### 4.1. Relation to Previously Used Stimulation Protocols

Several years ago we had introduced a form of passive stimulation called tactile coactivation where a patch of skin was repetitively stimulated to induce plastic changes in somatosensory cortex in parallel to changes of tactile perception [37]. The basic idea behind this design was to coactivate a large number of receptive fields on the tip of the index finger in a Hebbian manner in order to strengthen their mutual interconnectedness (see [35] for review). Since then a number of modifications have been implemented to increase magnitude and stability of effects while at the same time reducing the time of intervention [26, 38, 39, 43, 44]. 

**Figure 5:**
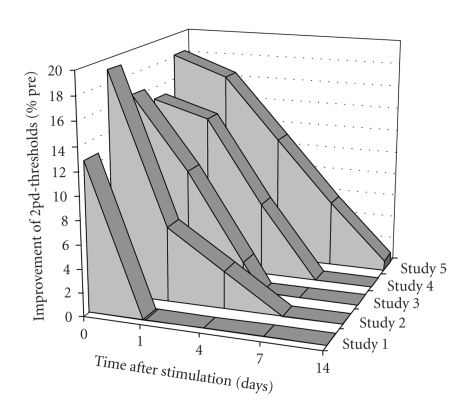
Stability of improvement of tactile acuity following different repetitive stimulation paradigms. The magnitude and the stability of the improvement of tactile acuity crucially depend on the stimulation protocol. Studies 1 [38], 2 [44], and 3 [43] were conducted in young subjects, whereas studies 4 [26] and 5 [the present work] were conducted in healthy elderly individuals. In studies 1, 2, and 4 an irregular stimulation was used for 3 hours, with stimuli drawn from a Poisson process and thresholded for a minimal interstimulus interval (ISI) of 100 ms, and maximal ISI of 3000 ms. Studies 3 and 5 used a high-frequency intermittent stimulation for 20 or 30 minutes, that consisted of stimulus-trains of 1 s (single pulse-duration: 0.2 ms @ 20 Hz) and intertrain intervals of 5 s. In studies 1 and 3, stimulation was applied to a single finger (index finger), while studies 2, 4, and 5 used multifinger stimulation (data of index finger is shown here). Study 5 featured electrical instead of tactile stimulation. Missing data points (study 3: “4days”, study 5: “1day”, “4days”) were linear interpolated.

When tactile coactivation was applied to a single finger for 3 hours, an improvement of tactile acuity of about 13% was reported that was reversible within 24 hours [38]. Extending the tactile coactivation to all fingers of a hand for 3 hours, we were able to evoke an average improvement of tactile acuity of about 20% immediately after application, of about 9% after 24 hours and of 5% after 96 hours [44]. These findings indicated synergistic effects of stimulating all fingers of a hand simultaneously. Later studies adapted intermittent high-frequency stimulation protocols usually used in experiments with brain slices to induce long-term potentiation of synaptic transmission. For example, applying 20 Hz trains of tactile stimuli once per second with intertrain intervals of 5 s to the tip of the index finger led to an improvement of about 16% after termination of stimulation and a significant improvement of 10% 24 hours later, indicating delayed recovery [43]. For a comparison of the outcome of different stimulation protocols on tactile acuity see Figure 5.

Evidence from fMRI and SEP measurements demonstrated that the underlying neural changes induced by coactivation are localized in somatosensory cortex [38–40]. Most importantly, combined assessment measuring tactile acuity and the cortical reorganization, it was found that the change in discrimination abilities could be predicted by the changes of the SEP-dipole localizations in SI [38, 40] or by changes in the cortical activation as measured as a blood oxygenation level-dependent (BOLD) signal using fMRI [39]. In all cases, the amount of perceptual gain resulting from this procedure linearly correlated with the amount of cortical reorganization suggesting a causal relation.

### 4.2. rES, Improvement of Sensorimotor Performance and Rehabilitation

Stimulation based on rES overcomes technical limitations associated with prior mechanical stimulation paradigms like limitations of stimulation intensity. Most importantly, rES activates Ia large muscle afferents, group Ib afferents from Golgi organs, group II afferents from slow and rapidly adapting skin afferents and cutaneous afferent fibers [48, 49], what is in contrast to tactile stimulation, which is basically transmitted via Meissner or Merkel receptors. This becomes particularly important when working with elderly individuals, as the number of mechanoreceptors decreases during aging [5–9], complicating the efficacy cutaneous stimulation. 

Besides investigations in healthy subjects [50–54] many attempts have been made to explore the efficacy of rES-like stimulation as therapeutical intervention in chronic stroke patients [55–66]. In these studies stimulation was applied according to various protocols. The duration of stimulation varied between 20 minutes and 2 hours per day, whereas the duration of the whole intervention ranged from a single application to 8 weeks of repeated application. The stimulation sequences in most cases used repetitive square pulses in a frequency range of 1 Hz to 100 Hz, with higher frequencies applied more often. A number of clinical methods (SEP, MEP, fMRI) and functional assessments of sensorimotor performance was used and revealed specific changes following the respective intervention. Wu and co-workers, for example, demonstrated the effects of electric stimulation applied to a paretic limb in chronic stroke patients. As a result, hand function as estimated by means of the Jebsen-Taylor Functional Hand Test improved significantly after stimulation [60]. These results are in accordance with our own findings obtained in chronic stroke patients [66] and acute stroke patients [64], where we found a significant improvement of tactile and sensorimotor performance including haptic object recognition and fine motor performance following a therapeutical intervention based on rES applied daily over several weeks. Our present data show that effects of rES can be maintained at least for up to 2 weeks. It appears conceivable to assume that longer periods of repeated application of rES would make the effects even more durable. In a recent study in chronic stroke patients individuals were treated with rES for 4 days a week for 6 weeks, which led to a significant improvement of sensory and motor performance of the affected hand. Remarkable, after a follow-up of 6 weeks, the same magnitude of beneficial effects of rES on sensorimotor performance could be recorded [66]. In another study on subacute stroke patients, daily application of rES over 4 weeks induced beneficial effects on sensorimotor performance that were preserved even after 3 months of follow-up [64]. Accordingly, there is converging evidence that rES-induced affects can be quite long-lasting.

Possible mechanisms of rES-like stimulation engaged in the sensorimotor improvement include facilitation of sensorimotor integration in the relevant brain networks by increasing cortical excitability and neural activity [67] which is in line with our findings based on tactile stimulation studies in humans [35, 38, 39]. Alternative interpretations refer to interhemispheric competition models for sensory and motor processing [68, 69] utilizing upregulation of excitability within the stroke-affected hemisphere or downregulation of excitability within the unaffected hemisphere [65]. However it has been demonstrated that rES-like paradigms in combination with motor training facilitate the beneficial effects of rehabilitative treatments [62], making these approaches prime candidates for future therapeutic interventions.

## 5. Conclusion

In our study we showed that repeated application of repetitive electric stimulation (rES) to all fingers of a hand in elderly subjects is a promising tool for the improvement of tactile acuity, haptic, and fine motor performance. The gain in performance was maintained throughout the observation window of 2 weeks after rES intervention. These findings corroborate earlier findings about a close interconnectedness of the somatosensory and motor system. Moreover, our data provide further support for the suitability of rES paradigms for maintenance of sensorimotor performance in elderly individuals, and its therapeutic potential for restoration of sensorimotor functions in patients.

## References

[1] Cua AB, Wilhelm K-P, Maibach HI (1990). Elastic properties of human skin: relation to age, sex, and anatomical region. *Archives of Dermatological Research*.

[2] Doubal S, Klemera P (1998). Changes in mechanical properties of skin as a marker of biological age. *Sbornik Lekarsky*.

[3] Woodward KL (1993). The relationship between skin compliance, age, gender, and tactile discriminative thresholds in humans. *Somatosensory and Motor Research*.

[4] Vega-Bermudez F, Johnson KO (2004). Fingertip skin conformance accounts, in part, for differences in tactile spatial acuity in young subjects, but not for the decline in spatial acuity with aging. *Perception and Psychophysics*.

[5] Cauna N, Mannan G (1958). The structure of human digital pacinian corpuscles (corpus cula lamellosa) and its functional significance. *Journal of Anatomy*.

[6] Bruce MF (1980). The relation of tactile thresholds to histology in the fingers of elderly people. *Journal of Neurology Neurosurgery and Psychiatry*.

[7] Cauna N, Montagna W (1987). The effects of aging on the receptor organs of the human dermis. *Advances in the Biology of the Skin*.

[8] Besne I, Descombes C, Breton L (2002). Effect of age and anatomical site on density of sensory innervation in human epidermis. *Archives of Dermatology*.

[9] Iwasaki T, Goto N, Goto J, Ezure H, Moriyama H (2003). The aging of human Meissner’s corpuscles as evidenced by parallel sectioning. *Okajimas Folia Anatomica Japonica*.

[10] Bolton CF, Winkelmann RK, Dyck PJ (1966). A quantitative study of Meissner’s corpuscles in man. *Neurology*.

[11] Quilliam TA, Ridley A (1971). The receptor community in the finger tip. *Journal of Physiology*.

[12] Bouche P, Cattelin F, Saint-Jean O (1993). Clinical and electrophysiological study of the peripheral nervous system in the elderly. *Journal of Neurology*.

[13] Caruso G, Nilsson J, Crisci C, Nolano M, Massini R, Lullo F (1993). Sensory nerve findings by tactile stimulation of median and ulnar nerves in healthy subjects of different ages. *Electroencephalography and Clinical Neurophysiology*.

[14] Rivner MH, Swift TR, Malik K (2001). Influence of age and height on nerve conduction. *Muscle and Nerve*.

[15] Valerio BC, Nobrega JA, Tilbery CP (2004). Neural conduction in hand nerves and the physiological factor of age. *Arquivos de Neuro-Psiquiatria*.

[16] Taylor PK (1984). Non-linear effects of age on nerve conduction in adults. *Journal of the Neurological Sciences*.

[17] Verdu E, Ceballos D, Vilches JJ, Navarro X (2000). Influence of aging on peripheral nerve function and regeneration. *Journal of the Peripheral Nervous System*.

[18] Peters A (2002). The effects of normal aging on myelin and nerve fibers: a review. *Journal of Neurocytology*.

[19] Raz N, Gunning FM, Head D (1997). Selective aging of the human cerebral cortex observed in vivo: differential vulnerability of the prefrontal gray matter. *Cerebral Cortex*.

[20] Sowell ER, Peterson BS, Thompson PM, Welcome SE, Henkenius AL, Toga AW (2003). Mapping cortical change across the human life span. *Nature Neuroscience*.

[21] Cabeza R, Anderson ND, Locantore JK, McIntosh AR (2002). Aging gracefully: compensatory brain activity in high-performing older adults. *NeuroImage*.

[22] Kalisch T, Ragert P, Schwenkreis P, Dinse HR, Tegenthoff M (2009). Impaired tactile acuity in old age is accompanied by enlarged hand representations in somatosensory cortex. *Cerebral Cortex*.

[23] Stevens JC (1992). Aging and spatial acuity of touch. *Journals of Gerontology*.

[24] Sathian K, Zangaladze A, Green J, Vitek JL, DeLong MR (1997). Tactile spatial acuity and roughness discrimination: impairments due to aging and Parkinson’s disease. *Neurology*.

[25] Tremblay F, Wong K, Sanderson R, Cote L (2003). Tactile spatial acuity in elderly persons: assessment with grating domes and relationship with manual dexterity. *Somatosensory and Motor Research*.

[26] Kalisch T, Tegenthoff M, Dinse HR (2008). Improvement of sensorimotor functions in old age by passive sensory stimulation. *Clinical Interventions in Aging*.

[27] Stevens JC, Patterson MQ (1995). Dimensions of spatial acuity in the touch sense: changes over the life span. *Somatosensory and Motor Research*.

[28] Stevens JC, Choo KK (1996). Spatial acuity of the body surface over the life span. *Somatosensory and Motor Research*.

[29] Dinse H, Wilimzig C, Kalisch T, Grunwald M (2008). Learning effects in haptic perception. *Human Haptic Perception: Basics and Applications*.

[30] Bock O, Schneider S (2002). Sensorimotor adaptation in young and elderly humans. *Neuroscience and Biobehavioral Reviews*.

[31] Sawaki L, Yaseen Z, Kopylev L, Cohen LG (2003). Age-dependent changes in the ability to encode a novel elementary motor memory. *Annals of Neurology*.

[32] Kornatz KW, Christou EA, Enoka RM (2005). Practice reduces motor unit discharge variability in a hand muscle and improves manual dexterity in old adults. *Journal of Applied Physiology*.

[33] Smith CD, Walton A, Loveland AD, Umberger GH, Kryscio RJ, Gash DM (2005). Memories that last in old age: motor skill learning and memory preservation. *Neurobiology of Aging*.

[34] Verbrugge LM, Jette AM (1994). The disablement process. *Social Science and Medicine*.

[35] Dinse HR, Kalisch T, Ragert P (2005). Improving human haptic performance in normal and impaired human populations through unattended activation-based learning. *ACM Transactions on Applied Perception*.

[36] Dinse HR, Kleibel N, Kalisch T, Ragert P, Wilimzig C, Tegenthoff M (2006). Tactile coactivation resets age-related decline of human tactile discrimination. *Annals of Neurology*.

[37] Godde B, Stauffenberg B, Spengler F, Dinse HR (2000). Tactile coactivation-induced changes in spatial discrimination performance. *Journal of Neuroscience*.

[38] Pleger B, Dinse HR, Ragert P, Schwenkreis P, Malin JP, Tegenthoff M (2001). Shifts in cortical representations predict human discrimination improvement. *Proceedings of the National Academy of Sciences of the United States of America*.

[39] Pleger B, Foerster A-F, Ragert P (2003). Functional imaging of perceptual learning in human primary and secondary somatosensory cortex. *Neuron*.

[40] Dinse HR, Ragert P, Pleger B, Schwenkreis P, Tegenthoff M (2003). Pharmacological modulation of perceptual learning and associated cortical reorganization. *Science*.

[41] Parsons CG, Danysz W, Quack G (1999). Memantine is a clinically well tolerated N-methyl-D-aspartate (NMDA) receptor antagonist—a review of preclinical data. *Neuropharmacology*.

[42] Walker-Batson D, Curtis S, Natarajan R (2001). A double-blind, placebo-controlled study of the use of amphetamine in the treatment of aphasia. *Stroke*.

[43] Ragert P, Kalisch T, Bliem B, Franzkowiak S, Dinse HR (2008). Differential effects of tactile high- and low-frequency stimulation on tactile discrimination in human subjects. *BMC Neuroscience*.

[44] Kalisch T, Tegenthoff M, Dinse HR (2007). Differential effects of synchronous and asynchronous multifinger coactivation on human tactile performance. *BMC Neuroscience*.

[45] Oldfield RC (1971). The assessment and analysis of handedness: the Edinburgh inventory. *Neuropsychologia*.

[46] Folstein MF, Folstein SE, McHugh PR (1975). “Mini mental state”: a practical method for grading the cognitive state of patients for the clinician. *Journal of Psychiatric Research*.

[47] Wickens TD (2002). *Elementary Signal Detection Theory*.

[48] Campbell W (1999). *Electrodiagnostic Medicine*.

[49] Kimura J (2001). *Electrodiagnosis in Diseases of Nerve and Muscle: Principles and Practice*.

[50] Golaszewski S, Kremser Ch, Wagner M, Felber S, Archner F, Dimitrijevic MM (1999). Functional magnetic resonance imaging of the human motor cortex before and after whole-hand afferent electrical stimulation. *Scandinavian Journal of Rehabilitation Medicine*.

[51] Golaszewski SM, Siedentopf CM, Koppelstaetter F (2004). Modulatory effects on human sensorimotor cortex by whole-hand afferent electrical stimulation. *Neurology*.

[52] Kaelin-Lang A, Luft AR, Sawaki L, Burstein AH, Sohn YH, Cohen LG (2002). Modulation of human corticomotor excitability by somatosensory input. *Journal of Physiology*.

[53] Ridding MC, Brouwer B, Miles TS, Pitcher JB, Thompson PD (2000). Changes in muscle responses to stimulation of the motor cortex induced by peripheral nerve stimulation in human subjects. *Experimental Brain Research*.

[54] Koesler IBM, Dafotakis M, Ameli M, Fink GR, Nowak DA (2008). Electrical somatosensory stimulation modulates hand motor function in healthy humans. *Journal of Neurology*.

[55] Powell J, Pandyan AD, Granat M, Cameron M, Stott DJ (1999). Electrical stimulation of wrist extensors in poststroke hemiplegia. *Stroke*.

[56] Conforto AB, Kaelin-Lang A, Cohen LG (2002). Increase in hand muscle strength of stroke patients after somatosensory stimulation. *Annals of Neurology*.

[57] Peurala SH, Pitkanen K, Sivenius J, Tarkka IM (2002). Cutaneous electrical stimulation may enhance sensorimotor recovery in chronic stroke. *Clinical Rehabilitation*.

[58] Wu CW-H, van Gelderen P, Hanakawa T, Yaseen Z, Cohen LG (2005). Enduring representational plasticity after somatosensory stimulation. *NeuroImage*.

[59] Sawaki L, Wu CW-H, Kaelin-Lang A, Cohen LG (2006). Effects of somatosensory stimulation on use-dependent plasticity in chronic stroke. *Stroke*.

[60] Wu CW-H, Seo H-J, Cohen LG (2006). Influence of electric somatosensory stimulation on paretic-hand function in chronic stroke. *Archives of Physical Medicine and Rehabilitation*.

[61] Celnik P, Hummel F, Harris-Love M, Wolk R, Cohen LG (2007). Somatosensory stimulation enhances the effects of training functional hand tasks in patients with chronic stroke. *Archives of Physical Medicine and Rehabilitation*.

[62] Conforto AB, Cohen LG, dos Santos RL, Scaff M, Marie SKN (2007). Effects of somatosensory stimulation on motor function in chronic cortico-subcortical strokes. *Journal of Neurology*.

[63] Ng SSM, Hui-Chan CWY (2007). Transcutaneous electrical nerve stimulation combined with task-related training improves lower limb functions in subjects with chronic stroke. *Stroke*.

[64] Dinse HR, Boland J, Kalisch T (2008). Repetitive sensory stimulation training in stroke. *European Journal of Neurology*.

[65] Koesler IBM, Dafotakis M, Ameli M, Fink GR, Nowak DA (2009). Electrical somatosensory stimulation improves movement kinematics of the affected hand following stroke. *Journal of Neurology, Neurosurgery and Psychiatry*.

[66] Smith PS, Dinse HR, Kalisch T, Johnson M, Walker-Batson D (2009). Effects of repetitive electrical stimulation to treat sensory loss in persons poststroke. *Archives of Physical Medicine and Rehabilitation*.

[67] Calautti C, Baron J-C (2003). Functional neuroimaging studies of motor recovery after stroke in adults: a review. *Stroke*.

[68] Liepert J, Hamzei F, Weiller C (2000). Motor cortex disinhibition of the unaffected hemisphere after acute stroke. *Muscle and Nerve*.

[69] Hummel FC, Cohen LG (2006). Non-invasive brain stimulation: a new strategy to improve neurorehabilitation after stroke?. *Lancet Neurology*.

